# Effect of Artificial Oocyte Activation on Intra-Cytoplasmic Sperm Injection Outcomes in Patients with Lower Percentage of Sperm Containing Phospholipase Cζ: A Randomized Clinical Trial

**Published:** 2019

**Authors:** Hamid Nazarian, Nahid Azad, Leila Nazari, Abbas Piryaei, Mohammad Hassan Heidari, Reza Masteri-Farahani, Maryam Karimi, Marefat Ghaffari-Novin

**Affiliations:** 1- Department of Biology and Anatomical Sciences, School of Medicine, Shahid Beheshti University of Medical Sciences, Tehran, Iran; 2- Department of Reproductive Biology, School of Medicine, Semnan University of Medical Sciences, Semnan, Iran; 3- Department of Obstetrics and Gynecology, Preventative Gynecology Research Center, Shahid Beheshti University of Medical Sciences, Tehran, Iran; 4- Urogenital Stem Cell Research Center, Shahid Beheshti University of Medical Sciences, Tehran, Iran; 5- Infertility and Reproductive Health Research Center, Shahid Beheshti University of Medical Sciences, Tehran, Iran

**Keywords:** Artificial oocyte activation, Biomarker, Intra-cytoplasmic sperm injection, Phospholipase C zeta, Sperm

## Abstract

**Background:**

Artificial oocyte activation (AOA) is a specialized method in assisted reproductive technique (ART). According to increasing concern about using AOA, it is necessary to evaluate sperm-borne oocyte activating factors (SOAFs) including phospholipase C zeta (PLCζ). In this study, PLCζ before AOA was evaluated first and then the impact of AOA on pre-implantation embryo development was investigated.

**Methods:**

This prospective clinical trial enrolled couples subjected to ICSI. By evaluating PLCζ, semen samples were categorized into two groups; I (Control) and II (PLCζ deficient). Retrieved oocytes from partners were put into three categories: control group (Injected with sperm from group I, n=113), group without AOA (Injected with sperm from group II and no exposure to AOA, n=106), and group AOA (Injected with sperm from group II and exposure to AOA, n=114). Finally, fertilization results were compared via Kruskal-Wallis followed by Dunn’s multiple comparison test. The p<0.05 was considered statistically significant.

**Results:**

Fertilization rate was significantly lower in the group without AOA compared to control group (41.9±6.3 *vs*. 78.1±4.7, p<0.001). AOA improved fertilization rate in group AOA compared to the group without AOA (69.5±3.9 *vs*. 41.9±6.3, p<0.01); however, cleavage (91.7±2.8, 90.9±4.6, and 95.2±3.4, respectively) and embryo quality (2.5±0.1, 2.3±0.2, and 2.4±0.2, respectively) scores were not substantially different between groups of control, with and without AOA.

**Conclusion:**

We showed that PLCζ can be considered as a good biomarker in evaluation of oocyte activation capability. Further studies are required to establish the best use of PLCζ as a biomarker in clinics.

## Introduction

Oocyte activation is an imperative stage in the initiation of embryo development during the fertilization. Indeed, the entrance of sperm into the oocyte causes sequences of calcium oscillations in its cytoplasm, regulating a series of molecular events such as exocytosis of cortical granules, recruitment of maternal mRNA, pronuclear formation, and polyspermy prevention. These events are collectively known as oocyte activation (1).

Based on the best available evidence in the literature, phospholipase C zeta (PLCζ) has been proposed to be the main factor involved in oocyte activation; however, this remains to be established definitively (2, 3). It has been suggested that PLCζ can start the calcium oscillations by hydrolysis of PIP2 into inositol-1,4,5-triphosphate (IP3) and diacylglycerol (DAG) after its entrance into the oocyte (4). Afterward, via the IP3 receptor (IP3R) on the surface of the endoplasmic reticulum produces calcium oscillations in oocytes (4, 5). Abnormal localization patterns, reduced amount of PLCζ, and total deficiency of protein in sperm fail to induce calcium oscillations (6–8), which can be rescued by microinjection of PLCζ mRNA as well as recombinant PLCζ protein into the oocyte (6, 7, 9).

Oocyte activation can be artificially induced by calcium ionophores. This method is known as artificial oocyte activation (AOA) and is believed to be useful in the case of fertilization failure that occurs in 1–5% of intra-cytoplasmic sperm injection (ICSI) cycles (5, 10, 11). However, AOA is not helpful for all cases of fertilization failure with a suspected oocyte-related activation deficiency (12). Although neonatal, developmental, and behavioral assessments of children born following ICSI-AOA revealed no serious adverse effects in a small sample size (13), AOA does not mimic accurately the physiological fertilization process which may have harmful effects on oocytes and embryos (14–16). Considering the increased awareness and extensive caution in the use of AOA (14, 17), it should only be used when it is defensible by supplementary data such as proven PLCζ deficiency (17).

Although PLCζ is suggested as a diagnostic biomarker for oocyte activation capability in prior studies (2, 5, 18–20), there is an ongoing need to establish reference ranges for its applying in infertility clinics. In this study, PLCζ was evaluated before AOA and then the impact of AOA on pre-implantation embryo development was investigated later.

## Methods

### Patient selection and ethics:

The study was reviewed and approved by the ethical committee of Shahid Beheshti University of Medical Sciences and appropriate regulatory bodies within the infertility center (Registration no. IR.SBMU.MSP. REC. 1395.93). The trial was registered at Iranian Registry of Clinical Trials (irct), number IRCT 2016 0722029027N4. Informed written consents for enrollment in the study were taken from all participants. Sperm samples were prepared from couples who had been referred to the infertility center and subjected to ICSI. After PLCζ evaluation, individuals who showed our inclusion criteria were enrolled in the study. The percentage of PLCζ positive sperm more than 85% (pos. PLCζ >85%) and the percentage of PLCζ positive sperm less than 60% (pos. PLCζ <60%) were the inclusion criteria for group I (Control) and group II (PLCζ deficient), respectively (8, 21). The exclusion criteria were any history of trauma and surgery of the testis, age >40 years, and having the percentage of PLCζ positive sperm between 85%-60% in men, and also any female factors of infertility, and age >35 years in women. The study was performed in two parts:

### Part I- Semen analysis and processing:

Semen samples were collected by masturbation after 2–5 days of abstinence. After the liquefaction of the semen, the sample was processed by swim-up technique (22). Briefly, after centrifuging the semen and washing, sperm plaque was overlayered by Ham’s F-10 medium (Sigma-Aldrich, Germany). After 30–45 *min*, sperm cells in the upper part of the medium were taken and used for ICSI. Conventional sperm parameters including sperm concentration (×10^6^/*ml*), total motility (Non-progressive+progressive spermatozoa) (%), and sperm morphology (%) were determined according to World Health Organization criteria and Kruger strict criteria (22, 23).

### PLCζ immunofluorescence staining in sperm:

As previously described (6, 24), PLCζ was identified using immunofluorescence staining with some alterations. First, sperm cells were fixed with freshly-prepared PBS/paraformaldehyde 4% for 10 *min* and permeabilized with 0.2% Triton x-100-PBS for 10 *min* on ice. After washing the sample with 0.1% Tween 20-PBS (PBST), blocking was performed with 5% normal goat serum (Sigma-Aldrich, Germany). Then, the sperm were incubated in primary antibody (A PLCζ Rabbit Polyclonal Antibody, LifeSpan BioSciences, USA) overnight at 4°*C*. After washing, the sperm were incubated with secondary antibody (Goat Anti-Rabbit IgG H&L, Abcam Company, UK) in the dark room. Finally, the cells were counterstained with DAPI (10 *μg/ml*) and mounted.

**Figure 1.**
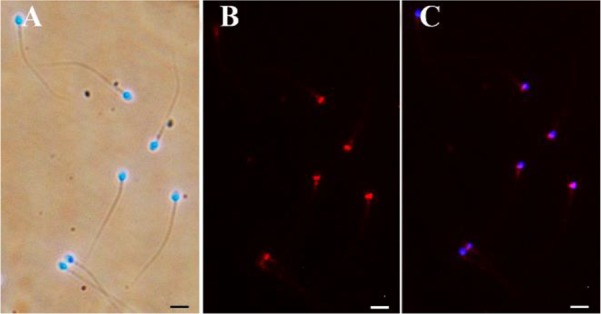
PLCζ immunofluorescence staining. The sperm nuclei are counterstained with DAPI. A: Merged DAPI and bright field images. B: Anti-PLCζ immunofluorescence. C: Merged DAPI and PLCζ stains. Bar=10 *μm*

Fluorescence images were captured with a Nikon microscope (TS100, Japan) equipped with a Nikon camera (DS-Fi1c, Japan) (Figure 1). Afterward, the percentage of sperm exhibiting PLCζ immunofluorescence was evaluated (n=200).

The mean percentage of PLCζ positive sperm less than 60% (pos. PLCζ <60%) was the inclusion criterion for PLCζ deficient group. For the control group, the percentage of PLCζ positive sperm more than 85% (pos. PLCζ >85%) was considered as the inclusion criterion (8, 21).

### Part II- Ovarian stimulation, ovum pickup, and IC-SI:

Recombinant FSH (Gonal-F, Merck Inc, Germany) was administered on menstrual cycle day 2–3 at an initial dose of 150 *IU/day*. During stimulation, transvaginal ultrasound was done. The gonadotropin dose was adjusted according to serial ultrasound monitoring. The GnRH antagonist (Cetritide, Merck, Germany) 0.25 *mg/day* was administered when the prominent follicle reached 14–15 *mm* in maximum diameter. When a minimum of two follicles reached a mean diameter of 18 *mm*, 10,000 *IU* of hCG (Choriomon, IBSA, Switzerland) was administered. Transvaginal oocyte retrieval was performed 36 *hr* after hCG administration. After retrieval, oocytes (n=359) were washed with HTF medium and put in CO_2_ incubator for 4 *hr*. Cumulus cells were removed mechanically and enzymatically by a 30-second exposure to hyaluronidase (Vitrolife, Sweden). Denuded oocytes were then evaluated for nuclear maturity. Oocytes with the first polar body were selected for ICSI. Eppendorf micromanipulator mounted on a Nikon inverted microscope was used to perform ICSI. Afterward, the injected oocytes were cultured in global medium (lifeGlobal, USA).

For each partner in group II (PLCζ deficient), a split ICSI-AOA cycle using sibling oocytes was performed. Next, the oocytes retrieved from each partner in group II were divided into two parts randomly (Groups AOA and without AOA). Collectively, oocytes were categorized into three groups: 1- group control (Injected with sperm from group I, n=113), 2- group without AOA (Injected with sperm from group II and no exposure to AOA, n=106), and 3- group AOA (Injected with sperm from group II and exposure to AOA, n=114).

### AOA:

Oocyte activation was performed as described by Montag et al. (25). Briefly, immediately after ICSI, oocytes were incubated in 10 *μmol/l* calcium ionophore A23187 for 15 *min* (Sigma-Aldrich, Germany) (Dissolved in DMSO) in culture medium covered by mineral oil. After washing with culture medium, injected oocytes were cultured in an incubator at 37*°C* under 6% CO_2_.

### Evaluation of fertilization rate (%), cleavage rate (%), and embryo quality score for each patient:

After 16–18 *hr* of post-injection, oocytes were observed for pronuclei observation. Oocytes failing to show 2 pronuclei after 25 *hr* were considered unfertilized. Fertilized oocytes were cultured in the incubator and their cleavage and quality was recorded on day 3 after oocyte retrieval. Blastomere number, size, and the percentage of fragmentation were assessed for embryo morphology (A, B, C). Grade A (Score 3) used for embryos with equal size of blastomeres and no fragmentation or less than 10%. Grade B (Score 2) was used for embryos with the even size of blastomeres and moderate fragmentation (Between 10–45%). Lastly, grade C (Score 3) was used for embryos with unequal size of blastomeres and severe fragmentation more than 45%. Embryo quality score of each patient was calculated as the sum of quality of scores for embryos/total number of cleaved embryos (26). The cleavage score was calculated as described by Sheikhi et al. (27). The cleavage score for each patient was calculated as the sum of cleaved embryos/total number of fertilized embryos (%).

### Statistical analysis:

All data were analyzed by Prism 6/Graph-Pad. Fertilization rates, embryo quality score, and cleavage scores were analyzed by Kruskal-Wallis test followed by Dunn’s multiple comparison test. Data were shown as a mean±standard error of the mean (SEM) in the text, figures, and tables. The p<0.05 was considered statistically significant.

## Results

### PLCζ deficiency:

The mean percentage of sperm exhibiting PLCζ immunofluorescence, and sperm parameters in the patients are presented in table 1. There was no significant difference in sperm concentration (×10^6^), total motility (%), and normal morphology (%) between groups. Eleven men showing the mean percentage of PLCζ positive sperm more than 85% entered group I (89±0.8) and 15 men showing the mean percentage of PLCζ positive sperm less than 60% entered group II (45.6±5.1). Among all individuals in group II, 6 men had at least one ICSI cycle with previous fertilization failure.

**Table 1.** Basic and demographic characteristics of couples studied

**Parameters**	**Group I (Control)** **(n=11)**	**Group II (PLCζ deficient)** **(n=15)**	**p-value**
**Female age (years)**	30.3±1.3	31.26±0.92	-
**Male age (years)**	32.1±1.2	33.06 ±0.98	-
**Sperm concentration (×10^6^/*ml*)**	53.6±9.6	31.2±8.06	0.06
**Total motility (Progressive + non-progressive) (%)**	53.2±7.5	37.6±6.5	0.1
**Sperm morphology (%)**	3.8±0.6	2.9±0.6	0.3
**The mean percentage of sperm containing PLCζ**	89±0.8	45.6±5.1	<0.0001

All data are shown as Mean±SEM

### ICSI outcomes:

**Figure 2.**
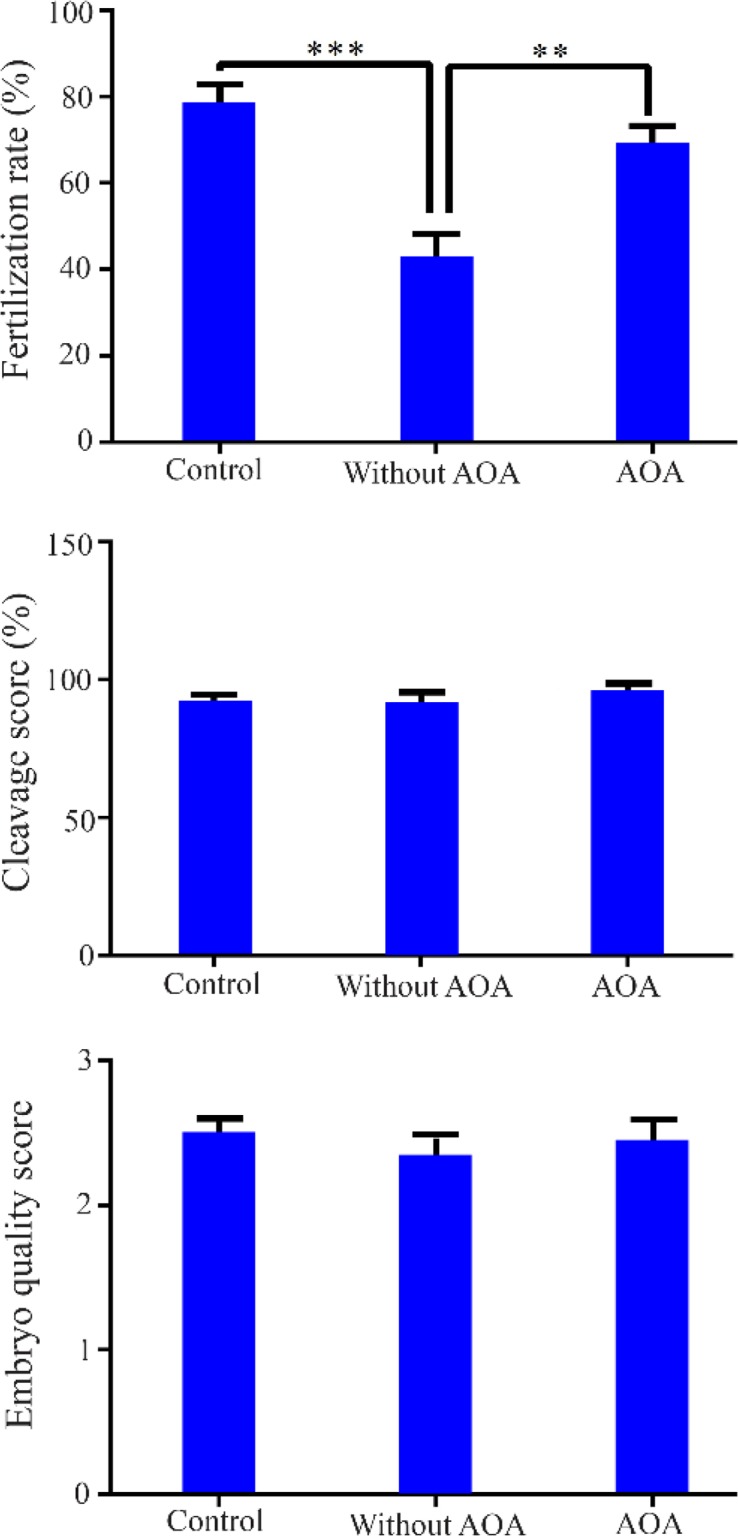
Comparison of fertilization rate, cleavage score, and embryo quality score between control, without AOA, and AOA groups. *** p<0.001 and **** p<0.0001, significant differences

The rate of fertilization, embryo quality, and cleavage scores were calculated on day 3 after ICSI. As shown in figure 2, significantly lower rate of fertilization was observed in group without AOA compared to control group (41.9±6.3 *vs*. 78.1±4.7, p<0.001), while incubation of oocytes in calcium ionophore in group AOA elicited a significant increase in fertilization rates compared to group without AOA (69.5±3.9 *vs*. 41.9±6.3, p<0.01). No significant difference in fertilization rate was observed between control and AOA groups (78.1±4.7 *vs*. 69.5±3.9, p>0.05). In addition, cleavage (91.7±2.8, 90.9±4.6, and 95.2±3.4, respectively) and embryo quality (2.5±0.1, 2.3±0.2, and 2.4±0.2, respectively) scores were not substantially different between groups of control, without AOA and AOA.

## Discussion

Our study showed that a lower percentage of sperm-containing PLCζ compared to control group causes a significantly lower rate of fertilization following ICSI, while the use of AOA can significantly improve that in these patients. It was also shown that embryo quality and cleavage scores between all groups (Control, without AOA, and AOA) did not differ significantly.

After fertilization, oocyte activation which is required for the development of the embryo occurs by a series of intracellular calcium oscillations (28). Calcium oscillations are believed to be triggered by PLCζ expression (18), affecting different aspects of oocyte activation and embryo development (29). Although PLCζ is reported to be a diagnostic biomarker for oocyte activation capacity (5, 18), further clinical studies are necessary to determine a reference range for the percentage of sperm-containing PLCζ affecting fertilization rates following ICSI. In this regard, Kashir et al. revealed a lower percentage of sperm-containing PLCζ in patients suffering from oocyte activation deficiency (OAD) compared to control group (27.4% in OAD group *vs*. 82.6% in control group) (8). It was also reported that patients with a history of fertilization failure show significantly lower percentages of PLCζ positive sperm (58.4±25) compared to the control group (87.6±9.8) (Mean±SD) (21). With considering these clinical experiments, individuals with the percentages of PLCζ positive sperm >85% as a control group (Group I) and also the percentages of PLCζ positive sperm <60% as PLCζ deficient group were included (Group II).

As shown in previous studies, PLCζ localization patterns, protein levels, and PLCζ positive sperm correlated with fertilization rates following ICSI (19, 20). In agreement with those studies, it was shown that fertilization rate was significantly lower in the group without AOA compared to control group. However, another study conducted by Ferrer-Vaquer et al. showed the opposite results, indicating no correlation between ICSI fertilization rates and PLCζ (30).

Several studies have been conducted on the application of AOA after conventional ICSI such as in patients with fertilization failure (12, 31), globozoospermia (32, 33), teratozoospermia (34, 35), and azoospermia or cryptozoospermia (36). However, to the best our knowledge, a few studies have been carried out on the use of AOA after PLCζ diagnostic tests in ICSI cycles in humans. Accordingly, Taylor et al. showed a lack of PLCζ in sperm from a globozoospermic patient using immunofluorescence and western blotting techniques. They found that ICSI followed by AOA resulted in high rates of fertilization and pregnancy in this patient (33). In another case-report study, PLCζ deficiency resulted in the failure of fertilization and impaired calcium oscillations during ICSI. AOA procedure following ICSI enhanced fertilization and development of the embryo in this patient (37). In a recent study, Tavalaee and Nasr-Esfahani indicated the lower expression of PLCζ and two other candidates for sperm born activating factors (SOAFs) [including postacrosomal sheath WW domain-binding protein (PAWP) and TR-KIT] in 12 globozoospermic men and observed an improvement in fertilization rate following ICSI-AOA in the majority of the patients (32). In accordance with these clinical studies, it was demonstrated that AOA significantly improved the rate of fertilization in patients with a lower percentage of sperm-containing PLCζ (Figure 2).

Our finding is similar to some animal studies which were performed in wobbler mice (A useful model for globozoospermia with abnormalities in localization, but not in PLCζ expression). Accordingly, microinjection of sperm from wobbler mice into oocytes led to lower fertilization rates, lower cleavage rate, and no blastocyst formation. The use of AOA in these oocytes recovered their fertilization ability, cleavage rate, and embryo development to blastocyst (38). In another study, Van-den Meerschaut et al. found that the percentage of oocytes injected with wobbler sperm showed less significant rises in calcium, lower fertilization rate, and lower development to the blastocyst stage compared with the wild type. Following AOA application, all of the mentioned parameters have been improved (39). However, these studies showing the effect of abnormal localization patterns and AOA on embryo cleavage and quality in wobbler mice manifested no considerable difference in terms of embryo quality and cleavage scores between our three groups. This result agreed with a recent study which showed no correlation between the percentage of PLCζ positive sperm and embryo quality (20). It seems that frequency and amplitude of calcium signals have no effect in early embryo cleavage of activated oocytes. However, the developmental capacity and morphology of post-implantation rabbit embryos were affected by abnormal calcium signals, suggesting the possibility of epigenetic events and long-term developmental effects (40). Similarly, Ozil et al. stated that the enhancement or reduction of calcium signaling following fertilization has no effect on the pre-implantation development of the embryo to blastocyst. Nevertheless, they confirmed long-term effects on both gene expression and development to term (29). These reports are in a good agreement with our results, showing similar embryo quality and cleavage in control, without AOA, and AOA groups although post-implantation development in our participants was not followed.

## Conclusion

In conclusion, our study confirmed previous studies reporting PLCζ as an oocyte activation factor that its deficiency can be amended by AOA. However, further studies with larger series are required to establish a reference value for the best clinical use of PLCζ and other SOAF(s) candidates in clinics as diagnostic biomarkers of oocyte activation.
